# Digitale Innovation in der Medizin – die COVID-19-Pandemie als Akzelerator von „digital health“

**DOI:** 10.1007/s41972-020-00126-2

**Published:** 2020-12-21

**Authors:** Jan-Niclas Mumm, Severin Rodler, Maja-Lena Mumm, Ricarda M. Bauer, Christian G. Stief

**Affiliations:** 1 Urologische Klinik und Poliklinik, Ludwig-Maximilians-Universität München, Campus Großhadern, Marchioninistraße 15, 81377 München, Deutschland; 2 Politik und Sozialwissenschaften, Institut für Publizistik und Kommunikationswissenschaften, Freie Universität Berlin, Berlin, Deutschland

**Keywords:** Digitalisierung, Digitale Gesundheit, Inkontinenz, Urologie, Digitalization, Digital health, Incontinence, Urology

## Abstract

Die COVID-19-Pandemie hat eine Welle der Digitalisierung in der Medizin ausgelöst. Der Einsatz modernster Technologien wird in den folgenden Jahren Routinediagnostik und Therapieansätze revolutionieren und die Arzt-Patienten-Beziehung positiv beeinflussen. Die Verwendung von AI („artifical intelligence“) und Big Data ist neben den Entwicklungen der mHealth („mobile health“) einer der bedeutendsten Meilensteine im Aufbau eines digitalen und intelligenten Gesundheitssystems.

## Abstract (en)

The COVID-19 pandemic has triggered a wave of digitalization in medicine. In the coming years, the use of state-of-the-art technologies will revolutionize routine diagnostic and treatment approaches, as well as have a positive effect on the physician–patient relationship. The application of AI (artificial intelligence) and Big Data, combined with the development of mHealth (mobile health), represents the most significant milestone in the creation of a digital and intelligent health system!

## Digitalisierung der Medizin

Das Aufkommen des Internets führte in der Gesellschaft zu einem großen Wandel, welcher jeden unserer Lebensbereiche beeinflusst; so auch die Medizin, und die Art und Weise, wie Gesundheitskommunikation gestaltet wird. Aufgrund der Orts- und Zeitunabhängigkeit des Internets sowie durch Mobiltelefone und Smartdevices entstanden neue Möglichkeiten für die Medizin bzw. die Gesundheitskommunikation. Diese Phänomene werden unter den Namen eHealth („electronic health“) und mHealth („mobile health“) diskutiert [1]. Das Gesundheitswesen wird durch diesen Wandel geradezu revolutioniert. Die Entwicklung von Gesundheits-Apps, Fitness-Apps oder Ernährungs-Apps nimmt immer mehr zu. Im Jahr 2019 wurden in der Rubrik „medical“ im App Store ca. 105.000 Apps und in der Rubrik „health&fitness“ ca. 210.000 Apps angeboten [2], wobei diese Zahl im letzten Jahr nochmals rapide zunahm. Das Spektrum von Gesundheits-Apps ist dabei sehr weit und kann von Fitnessübungen, Erinnern an Termine, Unterstützung bei der Ernährungsplanung, Übernahme organisatorischer Aufgaben, Messen wichtiger Werte, Überwachung von Krankheiten und Übernahme der Datenauswertung bis hin zur Diagnoseunterstützung oder sogar der Diagnosestellung reichen [3]. Auch Wearables wie z. B. Smartwatches werden immer häufiger zu gesundheitlichen Zwecken genutzt und können so als Kommunikationsgerät, zur Bestimmung von Vitalparametern wie EKG und Herzfrequenz oder zum Schlaftracking eingesetzt werden [4].

Eine besondere Unterkategorie der Gesundheits-Apps stellen echte Medizinprodukte dar. Medizinprodukte dürfen zu therapeutischen und diagnostischen Zwecken eingesetzt werden, was reine Fitness- oder nicht zertifizierte Gesundheits-Apps nicht dürfen. Für die Anwendung digitaler Medizinprodukte wurde in den letzten Jahren zunehmend Evidenz geschaffen. So konnte gezeigt werden, dass in der Neurologie mittels Smartphones der Schweregrad einer Parkinson-Erkrankung bestimmt werden kann, dass in der Kardiologie eine Detektion von Vorhofflimmern über Smartphones möglich ist und dass in der Behandlung von Kindern mit Aufmerksamkeitsdefizitsyndrom digitale Interventionen eingesetzt werden können [5–7]. Für die weitere Implementierung von digitalen Gesundheitslösungen ist allerdings noch ein großer Grundlagenforschungsaufwand notwendig.

Nach Prognosen der „Allied Market Research“ wird das weltweite Marktvolumen von mHealth im Jahr 2020 bei ca. 58,8 Mrd. US-Dollar liegen und hat sich damit seit dem Jahr 2017 verdoppelt [8].

## Situation in Deutschland

In Deutschland befindet diese Entwicklung vergleichsweise noch in den Startlöchern. So wurde im Jahr 2018 mit Hilfe des Digital-Health-Index der Grad der Digitalisierung des Gesundheitssystems ausgewählter EU- und OECD-Länder gemessen. Deutschland rangierte hier auf dem vorletzten Platz, während Estland, Kanada und Dänemark den höchsten Digitalisierungsgrad aufwiesen [9]. Das Potenzial für mHealth bzw. eHealth ist in Deutschland enorm, wird jedoch durch die mangelnde Digitalisierung des Gesundheitsbereichs, starre Reglementierungen sowie die Skepsis von Seiten der Ärzte als auch von Patienten ausgebremst [10]. Viele Krankenhäuser haben zwar teilweise mit der Digitalisierung ihrer Prozesse angefangen, diese aber nicht zu Ende gebracht. So ist es oft der Fall, dass Laborwerte schon digital vorliegen, dann jedoch ausgedruckt werden, um der Papierform der Patientenakte hinzugefügt zu werden, womit eine Hybridform entsteht [2]. Das Gesundheitssystem läuft oft 2‑spurig und wird dadurch sowohl für Patienten als auch für Ärzte sehr unübersichtlich. Eine weitere Digitalisierung in Krankenhäusern und Praxen und damit eine Verbesserung der Infrastruktur in diesem Bereich sind essenziell, um das Potenzial der neuen Technologien ausschöpfen zu können.

## Beschleunigung der Digitalisierung durch COVID-19

Die COVID-19-Pandemie kann hier als Akzelerator gesehen werden und spiegelt ebenfalls die Wichtigkeit dieser Entwicklung wider. Die steigende Relevanz und Potenziale in dieser Entwicklung nehmen immer größere Ausmaße an [11]. Laut einer Studie der Unternehmensberatung Roland Berger werden sich die digitalen Gesundheitsangebote bis ins Jahr 2025 verdoppeln, und dies nicht zuletzt durch den Effekt der COVID-19-Pandemie [12]. Zu Beginn der Pandemie wurde eine schnelle und unbürokratische Implementierung von „Remote“-Systemen zum Schutz der Patienten notwendig [13]. Im Rahmen dieser Implementierung wurde ein Prozess beschleunigt, der bereits im Rahmen des DVG (Digitale-Versorgungs-Gesetz) angestoßen worden war [14], im Rahmen der Pandemie aber pragmatisch weiterentwickelt wurde. Beispielsweise wurden die Krankschreibung per Telefon, die Onlinesprechstunde sowie die Abrechenbarkeit von Onlinesprechstunden für Kassenpatienten möglich gemacht. Diese neuen Entwicklungen und v. a. verbesserten rechtlichen Rahmenbedingungen müssen nun jedoch für die langfristige Nutzung in das Gesundheitssystem integriert werden. Diese neuen Möglichkeiten sollten nicht nur als kurzfristige Lösung für die Herausforderungen der Pandemie gesehen werden, sondern die dauerhafte Integration der digitalen Gesundheitsangebote sollte hier das Ziel darstellen. Die durch die Pandemie ausgelöste Welle der Digitalisierung sollte genutzt werden, um neue Lösungsansätze für die medizinische Versorgung zu integrieren.

## Artificial Intelligence in der Medizin

Eine weitere besondere Neuerung stellt die Entwicklung von AI („artifical intelligence“) im Zusammenhang mit mHealth in der Medizin dar. Die möglichen Vorteile von künstlicher Intelligenz und der damit zusammenhängenden Datenanalyse und maschinellem Lernen wurden in den letzten Jahren immer wieder stark diskutiert [15]. Artificial Intelligence wird als transformative Errungenschaft für den Gesundheitssektor betrachtet und soll zur Verbesserung des Wohlstands der Weltwirtschaft beitragen [16]. Künstliche Intelligenz kann als ein Prozess beschrieben werden, welcher durch Mathematik, Computerprogrammierung, Logik und Statistik die Datenanalyse unterstützen soll und damit zu Entscheidungsfindungen beiträgt [15]. Besonders durch die Entwicklungen der mHealth und die dadurch generierten umfangreichen Datensätze (Big Data) aus Gesundheitsprodukten muss eine Lösung für die Herausforderungen der Verarbeitung dieser Daten geschaffen werden [17]. Durch die Nutzung von mHealth entstehen extrem große Datensätze, die beispielsweise aus elektronischen Gesundheitsakten, medizinischen Bildern und anderen komplizierten Texten bestehen, welche nur schlecht interpretiert werden können bzw. bei welchen die Analyse durch den Menschen sehr viel Zeit in Anspruch nimmt [18]. Daher wird die Entwicklung der mobilen Gesundheitsangebote als Treiber für den Aufbau intelligenter Gesundheitssysteme betrachtet. Die AI soll soweit ausgebaut werden, dass Daten auf einer so hohen Ebene analysiert werden können, dass eine subjektive Reaktion des Menschen simuliert werden kann. Damit würde ein digitaler Experte entstehen, welcher beispielsweise Pathologieergebnisse automatisiert in einen Bericht umwandelt [15]. Die künstliche Intelligenz besitzt weiterhin die Fähigkeit, automatisch zu lernen, sich zu verbessern, ohne explizit dafür programmiert zu werden, also maschinell zu lernen [18]. Durch die Nutzung von Algorithmen kann ein solches Computersystem in kurzer Zeit weitaus mehr Informationen und auch Erfahrungen abrufen, als es für einen Menschen möglich ist [19].

Zurzeit wird AI am stärksten in der Radiologie genutzt. Hierzu wurden Bildverarbeitungsprozesse und Computeralgorithmen optimiert, um so eine schnelle Diagnose möglich zu machen und Informationen zur Verfügung zu stellen, die der Mensch übersehen hätte [19].

Doch auch in anderen Bereichen der Medizin wird die AI genutzt, um die Gesundheitsversorgung zu optimieren. Beispielsweise in der Diagnose von Brustkrebs, aber auch in vielen Bereichen der Kardiologie führt AI zu einer schnelleren Interpretation und Diagnose, auch die Diagnose und Behandlung in der Gastroenterologie werden durch AI verbessert, ebenso kommt es durch eine Nutzung von AI zu einer früheren Diagnose von diabetischer Retinopathie [19]. AI ist also schon in vielen Bereichen der Medizin im Einsatz und optimiert die Patientenversorgung. Neben diesen größtenteils im Bereich der automatischen Bilderkennung angesiedelten Bereiche, kann künstliche Intelligenz in Zukunft eingesetzt werden, um Diagnosen zu stellen oder Patienten zu beraten, was aktuell bereits in Form von Chatbots getestet wird [6].

Durch die neuen Entwicklungen von AI und die daraus entstehenden neuen Möglichkeiten für die Patientenversorgung ist es von großer Notwendigkeit, dass Ärzte lernen, mit diesen umzugehen. Aktuell ist künstliche Intelligenz nicht Teil des medizinischen Curriculums, sollte für einen effektiven Einsatz von AI in der Medizin aber zeitnah implementiert werden [7]. Die Risiken der Nutzung von AI müssen durch geschultes Personal minimiert werden, während das positive Potenzial dieser Entwicklung maximiert wird [16].

AI in der Medizin kann zu einer besseren Patientenversorgung führen, frühere Diagnosen stellen, klinische Arbeitsabläufe beschleunigen, und hierbei durch die Reduktion der Fehlerzahl Kosten einsparen [19]. Die Nutzung von AI und die Analyse von Big Data sind neben den Entwicklungen der mHealth die wichtigsten Meilensteine im Aufbau eines intelligenten Gesundheitssystems [18].

## Digitale Gesundheitsanwendung (DiGA) – App auf Rezept

Im Oktober dieses Jahres wurden in Deutschland die ersten Apps vom Bundesinstitut für Arzneimittel und Medizinprodukte (BfArM) geprüft und dürfen nun von Ärzten verschrieben werden und werden von gesetzlichen Krankenversicherungen erstattet [20]. Dies zeigt, dass das Umdenken auch in der deutschen Gesundheitsversorgung angekommen zu sein scheint. Das Umdenken bzw. die Digitalisierung werden von Patienten und Ärzten gefordert, ein neues Selbstverständnis mit dem Umgang von Technologien ist allgegenwärtig. Das Gesundheitssystem in Deutschland muss also nachziehen.

Die Apps sollen Nutzer bzw. Patienten dazu motivieren mehr Verantwortung für die eigene Gesundheit zu übernehmen, ihr Engagement soll gesteigert werden, indem sie sich in den eigenen Versorgungsprozess aktiv einbringen [3].

Um die neuen Möglichkeiten der Gesundheits-Apps nutzen zu können, ist es wichtig, dass Ärztinnen und Ärzte sich mit den neuen Technologien vertraut machen, damit sie ihre Patienten effektiv unterstützen können und die Möglichkeiten der Technologien ausgeschöpft werden. Denn nur durch eine intensive und sachkundige Betreuung der Patienten im Umgang mit mHealth kann deren Nutzung erfolgreich sein [10]. Vor allem die Nutzungsmotivation hat einen besonderen Stellenwert im Zusammenhang mit den Erfolgschancen von Gesundheits-Apps. Nur wenn Patienten die Apps regelmäßig und über einen längeren Zeitraum nutzen, können sie einen Mehrwert für das traditionelle Gesundheitsmanagement liefern [10]. Eine Nutzungsmotivation, die mittlerweile viel in Studien untersucht wurde und zu positiven Ergebnissen kam, ist die Implementierung von Gaming-Aspekten in den Gesundheits-Apps [10]. Durch Anreize dieser Art sollen v. a. jüngere Generationen zur Nutzung von Gesundheits-Apps motiviert werden.

## Alter ist kein Hinderungsgrund für die Nutzung

**Figure 1:**
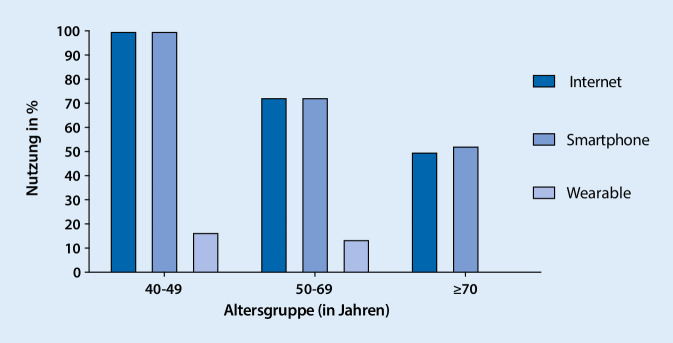

Jedoch sind die neuen Technologien auch besonders für die älteren Generationen geeignet. So werden beispielsweise Sprachassistenten über Smartphones von Menschen über 65 Jahren überproportional häufig genutzt [2]. Die digitalen Notrufsysteme, welche beispielsweise über einen Sturzalarm verfügen und per GPS den genauen Standpunkt der Person senden können, ist v. a. für die ältere Generation sehr hilfreich [2]. Im Jahr 2019 wurde ebenfalls ein „home care robot“ vorgestellt, welcher mit medizinischen Messgeräten vernetzt ist und so u. a. die Medikamenteneinnahme unterstützt und den Patienten überwacht [2]. Die neuen Entwicklungen werden also immer umfangreicher und können das Gesundheitssystem in vielen Bereichen unterstützen. Für die Nutzung digitaler Technologien ist der Zugang zu digitaler Infrastruktur wie Smartphones oder Wearables essenziell. Bereits jetzt ist eine starke Nutzung bis in hohe Altersgruppen zu beobachten, wie eine Studie aus unserer Klinik bei Patienten mit uroonkologischen Erkrankungen zeigte ([13]; Abb. 1).

## „Digital health“ in der Urologie

Auch in der Urologie finden digitale Technologien vermehrt Anwendung. So konnte gezeigt werden, dass die Behandlung von Harnwegsinfekten über Telemedizinplattformen möglich ist [21]. Eine breitere Anwendung zeigt sich jedoch für nicht ganz so zeitkritische Erkrankungen, sodass gerade im Bereich erektiler Dysfunktion durch Telemedizin v. a. in den USA Start-ups viele Patienten erreichen [22]. Neben telemedizinischen Lösungen liegt ein Schwerpunkt der aktuellen Anwendungen auf Patientenedukation, wobei viele verfügbare Apps noch kein standardmäßig hohes Niveau erreichen [23]. Am Beispiel Urolithiasis lässt sich zudem zeigen, dass Smartphone-Applikationen sogar teilweise schädlich sein können, wenn sie nicht medizinisch korrekten Inhalt gewährleisten [24]. Daneben wurden an der Schnittstelle zur Urogynäkologie in der Inkontinenzbehandlung der Frau zahlreiche digitale Behandlungsformen von Beckenbodentraining erprobt [25], wobei in hochmotivierten Probanden bessere Behandlungserfolge erzielt werden [26]. Perioperativ kann Aktivitätstracking eingesetzt werden, um Aktivitätsniveaus beispielsweise für Patienten zu erfassen, die sich einer Prostatektomie unterziehen [27]. Viele dieser Lösungen stellen aktuell Insellösungen dar und lassen sich noch nicht optimal in das Gesundheitssystem integrieren.

Zudem fehlen aktuell noch Leitlinien, welche aufzeigen, welche Indikationen wie digital behandelt werden können.

## Fazit für die Praxis

- Die Digitalisierung der Medizin hat weitreichende positive Effekte.
- Aufgrund der neuen Möglichkeiten können Patienten effektiv in ihren Versorgungsplan eingeschlossen werden und durch Selbstmanagement ihren Behandlungsplan straffen, wodurch wiederum Kosten reduziert werden können. Es kommt somit zu einer besseren Koordination von Leistungen, Verbesserung von Ressourcenmanagement, und es erleichtert den Zugang zu Spezialisten.
- Auch in der Urologie sind die ersten Ansätze von Telemedizin und dem Einsatz von „digital therapeutics“ vielversprechend.
- Für die optimale Behandlung von Patienten sollte jedoch noch mehr Evidenz geschaffen werden, an welcher Stelle der Einsatz digitaler Technologien eine echte Verbesserung der Versorgung bringt.
